# High-Throughput Fabrication of Quality Nanofibers Using a Modified Free Surface Electrospinning

**DOI:** 10.1186/s11671-017-2240-4

**Published:** 2017-07-26

**Authors:** Zhongbiao Shao, Liang Yu, Lan Xu, Mingdi Wang

**Affiliations:** 1 National Engineering Laboratory for Modern Silk, College of Textile and Clothing Engineering, Soochow University, 199 Ren’ai Road, Suzhou, 215123 China; 2 School of Mechanical and Electric Engineering, Soochow University, 178 Ganjiang Road, Suzhou, 215021 China

**Keywords:** Electrospinning, Polymers, Nanofibers, Bubbles, High throughput fabrication

## Abstract

Based on bubble electrospinning (BE), a modified free surface electrospinning (MFSE) using a cone-shaped air nozzle combined with a solution reservoir made of copper tubes was presented to increase the production of quality nanofibers. In the MFSE process, sodium dodecyl benzene sulfonates (SDBS) were added in the electrospun solution to generate bubbles on a liquid surface. The effects of applied voltage and generated bubbles on the morphology and production of nanofibers were investigated experimentally and theoretically. The theoretical analysis results of the electric field were in good agreement with the experimental data and showed that the quality and production of nanofibers were improved with the increase of applied voltage, and the generated bubbles would decrease the quality and production of nanofibers.

## Background

Electrospinning has been recognized as a simple and efficient technique for the production of polymer nanofibers. Due to the high surface area, high surface energy, and high surface activity et al., electrospun nanofibers can be used in a wide variety of applications such as nonwoven fabrics [1], reinforced fibers [2], drug delivery systems [3], tissue engineering [4], fuel cells [5], composites [6], filtration [7], photonics [8], sensorics [9], supercapacitors [10], wound dressing [11], and so on [12–15].

Conventional single-needle electrospinning inhibits the application of nanofibers to commercial applications due to its low production, usually at the level of 0.01–0.1 g/h [16]. It is desirable to obtain massive production of quality nanofibers to broaden the applications of nanofibers. Many efforts have concentrated on enhancing the production of the electrospinning technique. Ding et al. [17] successfully spun fibers by using a multi-needle electrospinning system. Dosunmu et al. [18] developed an electrospinning technique equipped with a porous tube. Yarin et al. [19] presented a free surface electrospinning (FSE) for mass production of nanofibers based on combination of normal magnetic and electric fields acting on a two-layer system. Jirsak et al. [20] patented a FSE using a rotating horizontal roller as the nanofiber generator. Wang et al. [21] demonstrated a novel needleless electrospinning using a conical metal wire-coil as spinneret. Lu et al. [22] reported a new high-throughput electrospinning technique with a large metal rotating cone as the spinneret. Qin et al. [23] presented a FSE setup using one-stepped pyramid-shaped copper spinneret to form multiple jets. Chen et al. [24] employed a gas pump to generate bubbles on a liquid surface to produce multiple jets. Liu et al. [25] proposed an electrospinning technique using needle-disk as spinneret to enhance the nanofiber throughput. In addition, numerical simulations for nanofluid [26] were presented to research the dynamics of charged jets. And the effects of various parameters, such as electric field [27] and magnetic field [28], on behavior of nanofluid, were systematically carried out.

In this paper, a modified free surface electrospinning (MFSE) using a cone-shaped air nozzle combined with a solution reservoir made of copper tubes was presented to obtain high throughput fabrication of quality nanofibers based on bubble electrospinning (BE) [24]. The nozzle combined with the solution reservoir made of copper tubes was used to produce multiple jets to initiate the electrospinning process. The effectiveness of the MFSE was experimentally studied by measuring the diameter distribution and throughput of nanofibers. The results showed that the quality and production of nanofibers were improved with the increase of applied voltage. Compared with the BE, the MFSE could produce nanofibers under a much higher applied voltage which would result in decreasing the nanofiber diameter, enhancing the diameter distribution, and improving the nanofiber throughput.

Surface-active agents are generally used to decrease the surface tensions of polymer solutions, which significantly affect the generation of bubbles. As a result, the formation and stabilization of bubbles are greatly dependent on the composition and physicochemical properties of the surface-active agents used [29]. Previously, we found that even just a bit of sodium dodecyl benzene sulfonate (SDBS), a surface-active agent, could significantly reduce the surface tension, facilitate the spinning process, and improve mechanical properties of electrospun polyvinyl alcohol (PVA) nanofibers [30]. Therefore, SDBS was added in the electrospun solution to generate bubbles on a liquid surface in this study. The effect of bubbles on the morphology and production of nanofibers was investigated experimentally and theoretically. The theoretical analysis results of electric field were in good agreement with the experimental data and showed that the bubbles would decrease the quality and production of nanofibers.

## Methods

### Materials

PVA with 1750 ± 50° of polymerization and SDBS were purchased from Sinopharm Chemical Reagent Co., Ltd. (Shanghai, China). PVA aqueous solutions with the concentration of 7 wt% were prepared by dissolving PVA powder in deionized water. And 0.3 wt% SDBS was dissolved in the PVA solutions. Then, the solutions were stirred at 90 °C for 2 h until it became homogeneous. All chemicals were of analytical grade and used without further purification.

### MFSE Apparatus

**Fig. 1:**
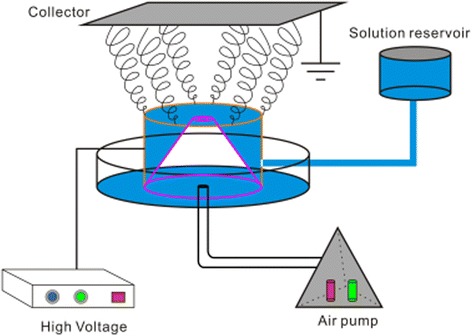
Schematic of the MFSE apparatus

The schematic of the MFSE apparatus was represented in Fig. 1. The apparatus consisted of a variable high-voltage power generator (0–150 kV, TRC2020, Dalian Teslaman Technology Co., LTD), a gas pump (TEION4500co, Eiko, Japan), a right circular cone-shaped air nozzle with a gas tube, a vertical solution reservoir made of copper tubes with the inner diameter 40 mm and the height 30 mm, and a grounded collector over the reservoir. The height of the cone-shaped air nozzle was 20 mm; the inner diameter of its base was 40 mm and that of its top was 1.5 mm. The nozzle was made of polyethylene (PE), and its top should be flush with the top of the copper solution reservoir. The positive terminal of the power generator was directly connected to the solution reservoir. The voltage supplied by the power generator was designated as the spinning voltage.

However, the solution reservoir of the BE apparatus was made of polymer tubes. And a thin polymer tube as nozzle was fixed on the center of the reservoir bottom. A slim metal needle, as the positive electrode, went through the nozzle. The nozzle and the needle were inserted through the bottom of the reservoir and connected with the gas pump and the generator, respectively. The metal electrode would lead to lower applied voltage. Compared with the BE, the MFSE could produce nanofibers under a much higher applied voltage which would result in improving the nanofiber throughput.

### MFSE Process

According to Ref. [23, 29] and our previous work [30], the electrospinning parameters were set as follows: PVA concentration 7 wt%, SDBS concentration 0.3 wt%, the applied voltage varied from 30 to 70 kv, and the working distance from the nozzle to the grounded collector was 13 cm. The MFSE experiments were carried out at room temperature (20 °C) at a relative humidity of 60%.

**Fig. 2:**
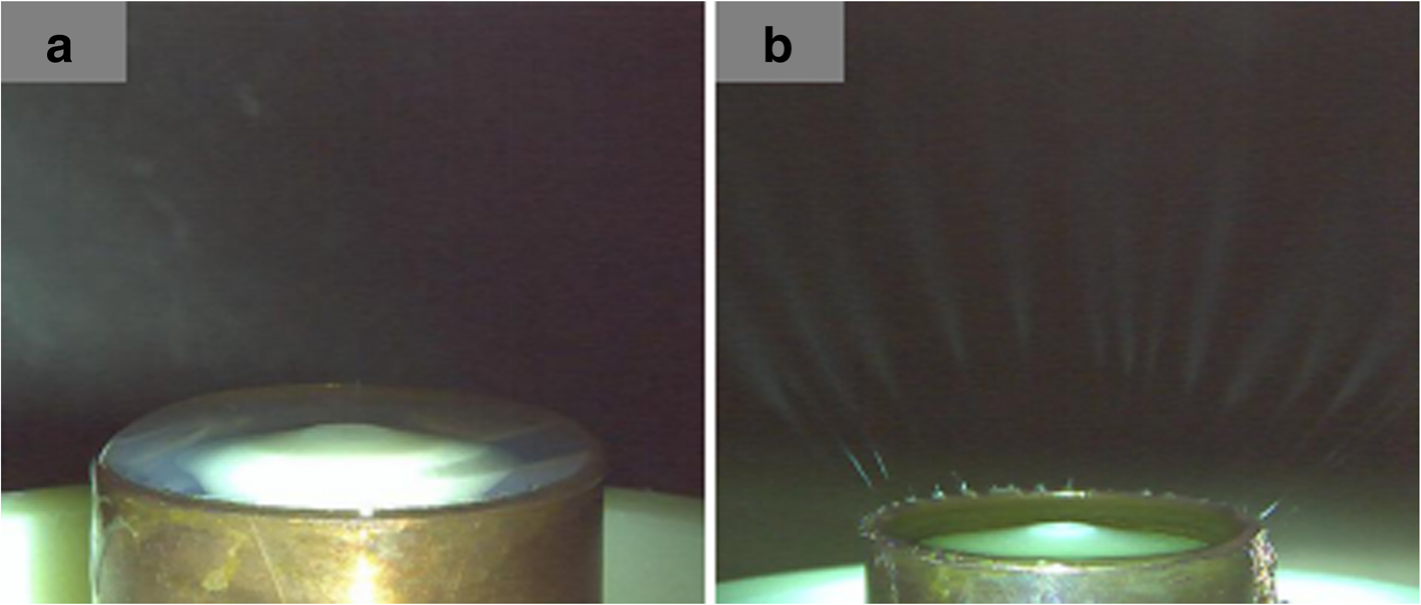
Photograph of the MFSE without SDBS. **a** Photograph of liquid surface and **b** photograph of spinning process

The PVA solution was poured into the reservoir, and the liquid surface was higher than the nozzle. Turning on slowly the gas valve, the liquid surface forms a bow around the nozzle due to the high surface tension of the solution. After an electronic field was applied and the voltage was over the threshold voltage, multiple jets initiated at the convex liquid surface, see Fig. 2.

**Fig. 3:**
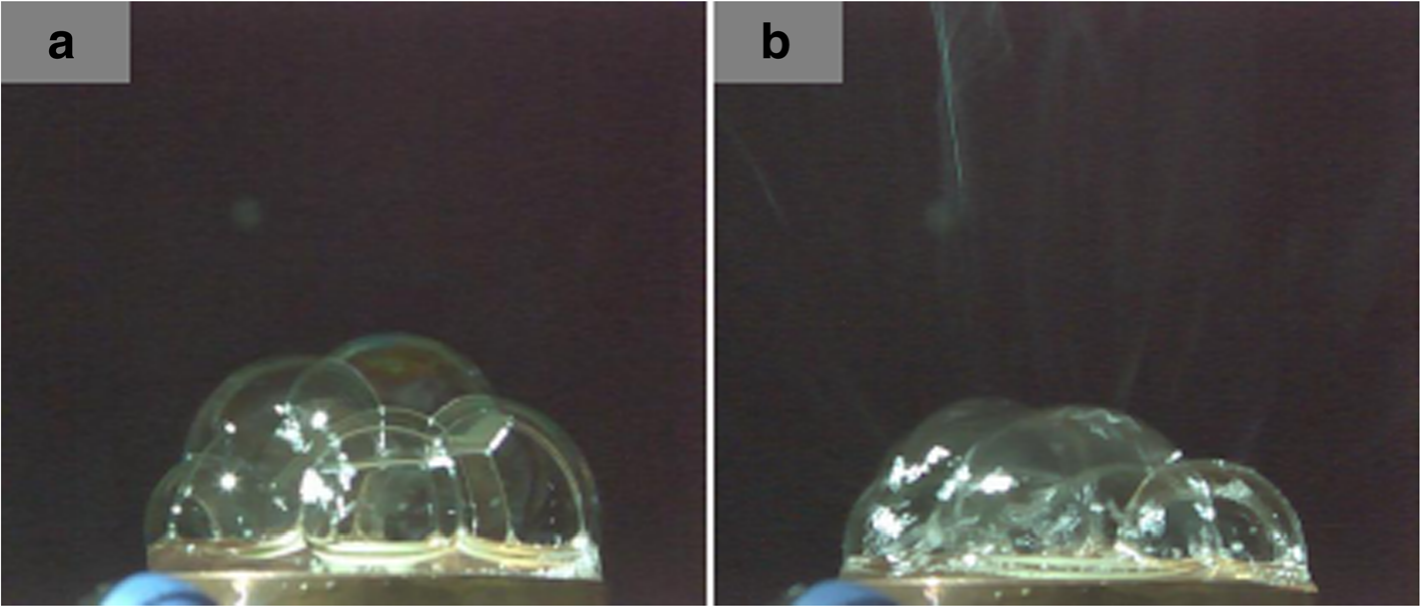
Photograph of the MFSE with the addition of SDBS. **a** Photograph of liquid surface and **b** photograph of spinning process

Several bubbles, whose diameters varied from 10 to 30 mm, were generated at the free surface of the polymer solution with the addition of SDBS. These bubbles would be broken into very small ones on their surface. When the surface tension of the small bubbles reduced to the critical value which could be overcome by the applied electric field, multiple jets were ejected from the bubbles to the collector, see Fig. 3.

### Measurements and Characterizations

The motion of the jets was observed by a high-definition camera at a frame rate of 25,000 frames/s (KEYENCE, VW-9000, Japan). Diameter and arrangement of electrospun PVA nanofibers were characterized using a scanning electron microscopy (SEM; Hitachi S-4800, Japan). All samples were dried at room temperature and then sputter-coated with gold by an IB-3 (Eiko, Japan) for 10 min. The matrix morphology and fibrous diameter characterization were carried out using Image J software (National Institute of Mental Health, USA). The electric field distributions were calculated by the Maxwell 2D (ANSOFT Corporation, USA).

## Results and Discussion

### Effect of Applied Voltage on the PVA Nanofibers

**Fig. 4:**
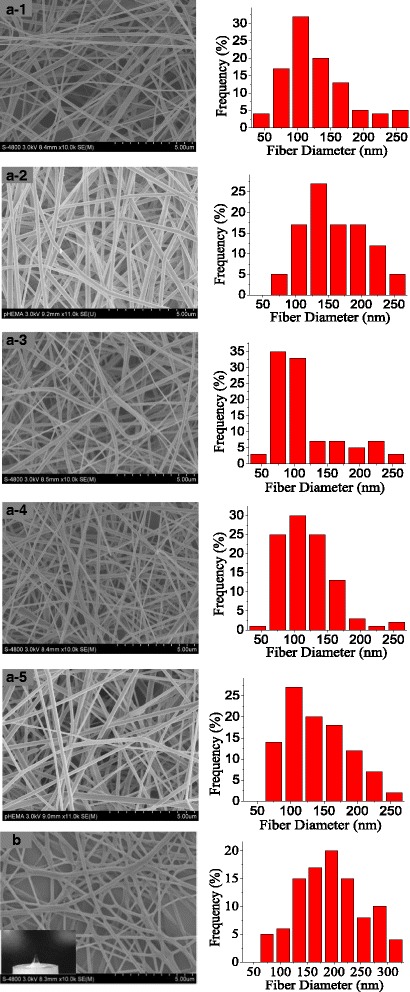
SEM images of PVA nanofibers. **a** MFSE with different applied voltage, (**a-1** 30 kV, **a-2** 40 kV, **a-3** 50 kV, **a-4** 60 kV, **a-5** 70 kV);. **b** BE (30 kV). *Inset*: a photograph of BE process. The right figures were the according diameter distribution

The morphologies of PVA nanofibers obtained using MFSE and BE were respectively carried out by SEM. SEM images and the according diameter distribution of nanofibers with different applied voltages in MFSE process were shown in Fig. 4a, and those of BE were indicated in Fig. 4b. When the applied voltage was 30 kV, the average diameter of nanofibers obtained by MFSE was 148 ± 8.53 nm and that of BE was 190 ± 8.26 nm. It could be seen that the PVA nanofibers produced by MFSE were finer and more homogeneous than those of BE. And the diameter distribution was more homogeneous with the increase of the applied voltage in MFSE process.

**Fig. 5:**
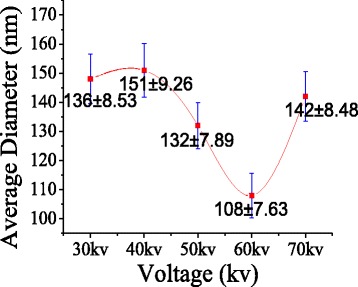
The effect of applied voltage on the average diameter

**Fig. 6:**
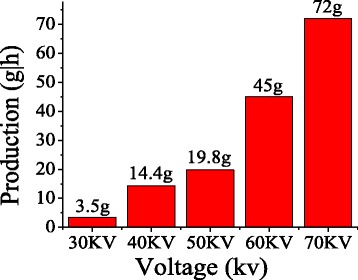
The production with the different applied voltage using MFSE

Figures 5 and 6 show the effect of applied voltage on the average diameter and production of the PVA nanofibers prepared by MFSE. It was evident that when the applied voltage was lower than 30 kV, very few jets were generated as the electric force was not enough to overcome the surface tension. However, when the applied voltage was 70 kV the electric force produced would accelerate quickly the upward movement of the jets. And the highly accelerated upward movement would not further stretch the jet into smaller fibers. Therefore, with the increase of the applied voltage in MFSE process, the average diameter decreased firstly and then increased, and the production increased. It was obvious that the applied voltage played a crucial role in MFSE process, which directly affected the nanofiber production.

However, the solution reservoir in which a metal electrode fixed was made of polymer tubes in the BE setup. The positive terminal of the power generator was connected to the metal electrode which led to lower applied voltage. Therefore, the production of nanofibers prepared by BE was only 3 g/h [24].

### Effect of SDBS on the PVA Nanofibers

**Fig. 7:**
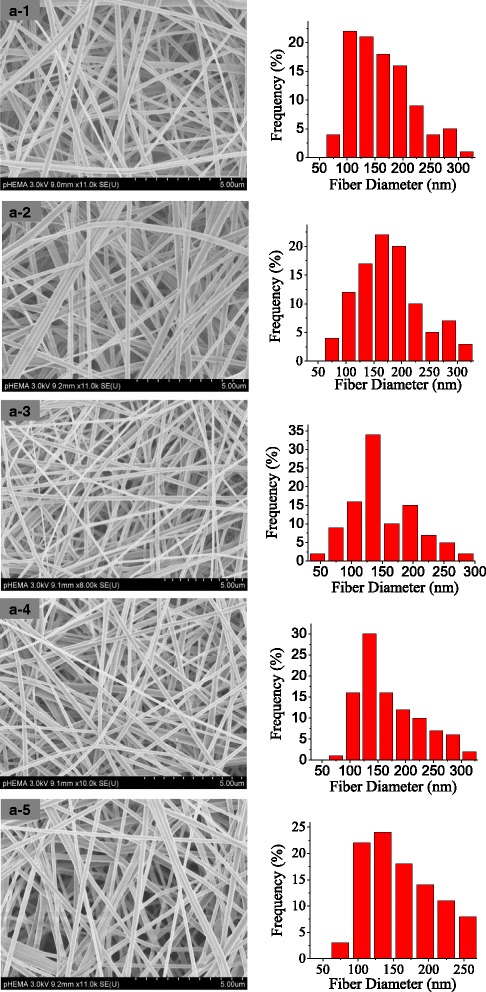
SEM images of PVA nanofibers prepared by MFSE at the different spinning time (**a-1** 5 min, **a-2** 10 min, **a-3** 15 min, **a-4** 20 min, **a-5**: 25 min). The right figures were the according diameter distribution

**Fig. 8:**
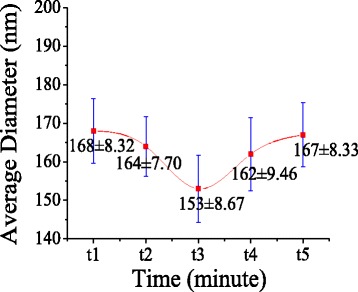
The effect of spinning time on the average diameter of PVA nanofibers prepared by MFSE

In the MFSE process, the addition of SDBS to PVA solution could effectively decrease the surface tension of the solution and generate bubbles on a liquid surface. Table 1 showed the increased electric conductivity and decreased surface tension of the PVA solutions with the addition of SDBS. Figures 7 and 8 illustrated SEM images and the according diameter distribution of nanofibers obtained from PVA solution with 0.3 wt% SDBS at an applied voltage of 60 kV with spinning time. It could be seen that the advantage diameter of nanofibers produced from PVA solution with 0.3 wt% SDBS were larger than those of nanofibers produced from pure PVA solution. And with the increase of spinning time, the MFSE made the diameter distribution of the PVA nanofibers to have little change, and the production of the PVA nanofibers was 12.5 g/h. The results showed the generated bubbles would increase the nanofiber diameter and decrease the nanofiber production. It was probably because of bubble formation, deformation, and break-wasted energy which could be used to further stretch the jet into smaller fibers. Moreover, the energy loss could make the charged jets move slower during the MFSE process, and the nanofiber production decrease.

**Table 1:** Surface tension and electrical conductivity of PVA solution without and with SDBS

SDBS (wt%)	Surface tension (mN/m)	Electric conductivity (us/cm)
0	45	8.8
0.3	33	43

The mechanical properties, such as tensile strength and elongation-at-break, of PVA nanofiber membranes without and with SDBS were shown in Table 2. It was seen that both the tensile strength and elongation-at-break of nanofiber membranes increased with the addition of SDBS. That meant the addition of SDBS could improve the mechanical properties of PVA nanofiber membranes.

**Table 2:** Mechanical properties of PVA nanofiber membranes without and with SDBS

SDBS (wt%)	Thickness (mm)	Tensile strength (MPa)	Elongation (%)
0	0.11 ± 0.01	7.58	6.44
0.3	0.10 ± 0.02	9.58	95.43

### Theoretical Analysis

Since the electric field is the main driving force to generate jets [23], the jet initiation is determined by the electric field intensity and the areas with higher electric field intensity generated jets more easily [25]. In order to reveal the experimental phenomenon, the electric field distributions around the free surface and the bubbles were calculated respectively by the Maxwell 2D.

**Fig. 9:**
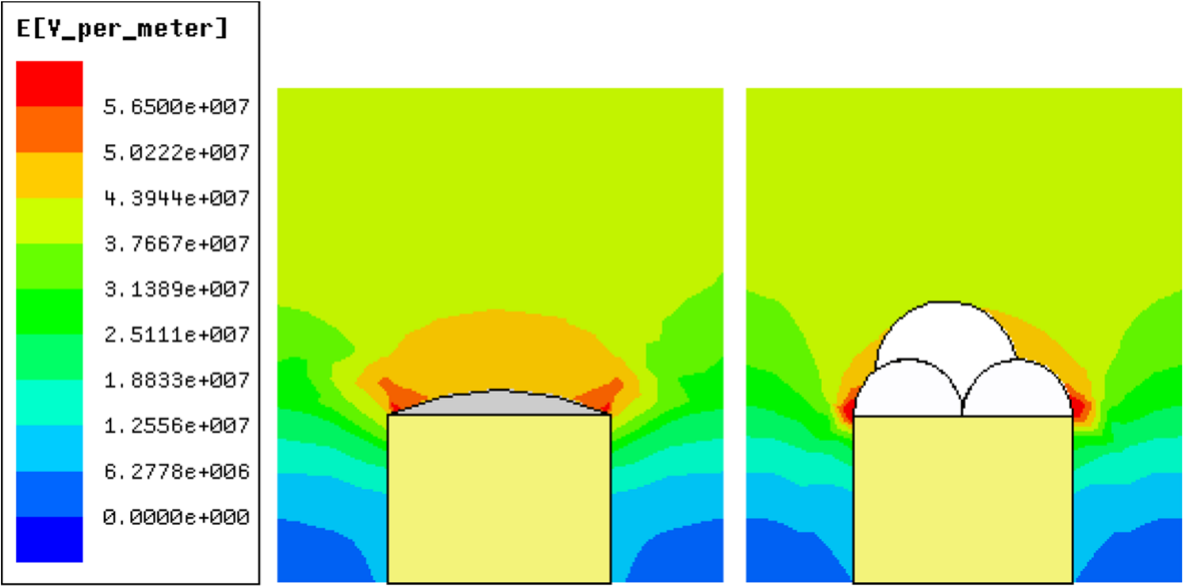
Simulation of electric field distributions at 60 kV (working distance 13 cm). **a** around the free surface. **b** around the bubbles

Figure 9 shows the simulation results of electric field distributions around the free surface and the bubbles with a working distance of 13 cm and an applied voltage of 60 kV. For the investigated MFSE process, the 2D simulations shown were performed for the following process parameters: the copper reservoir as positive pole was a rectangle with the width 40 mm and the height 30 mm, the electric conductivity of copper was 5.8 × 10^11^ us/cm, the working distance was 130 mm, the applied voltage was 60 kV, and the diameters of bubbles were 20 and 25 mm, the surface tensions of 7 wt% PVA solutions without and with SDBS were 45 and 33 mN/m, and the electric conductivity of these solutions were 8.8 and 43 us/cm respectively.

Figure 9a explores that the electric field on the curving part of the free surface was very heterogeneous and high, suggesting that those sites should be more efficient to self-generate jets. And the edge of the solution reservoir had much higher electric field intensity than the free surface due to metal materials of the reservoir. As shown in Fig. 2, a number of jets initiated around the curving part of free surface. And the higher electric field intensity around the edge rather than the surface could be the main reason that jets generated firstly from the edge of the solution reservoir. However, multiple jets were generated from the bubbles, as shown in Fig. 3. Fig. 9b reveals that the bubbles had lower electric field intensity than the curving part of the free surface. Since the electrostatic force was the main driving force to accelerate the movement of the jets, the jets would travel faster under a higher electric field. Therefore, the higher electric field on the curving part of the free surface than the bubbles could be the main reason that the generated bubbles would increase the nanofiber diameter and decrease the nanofiber production. The theoretical analysis results were in good agreement with the experimental results.

## Conclusions

In this paper, a high-effect free surface electrospinning using a cone-shaped air nozzle combined with a solution reservoir made of copper tubes was successfully developed to obtain high throughput fabrication of quality nanofibers for a long spinning time. The effects of the applied voltage on nanofiber quality and production were systematically investigated, and the results showed the quality and production of nanofibers were improved with the increase of applied voltage. Compared with the BE, the MFSE could produce nanofibers under a much higher applied voltage which would result in decreasing the nanofiber diameter, enhancing the diameter distribution, and improving the nanofiber throughput.

In addition, a surface-active agent, SDBS, was added in the electrospun solution to generate bubbles at the free surface of the solution in the MFSE process. The effect of bubbles on the nanofiber morphology and production was experimentally investigated. The results showed that with the increase of spinning time, the MFSE made the diameter distribution of the PVA nanofibers to have little change, and the generated bubbles would decrease the quality and production of nanofibers. Finally, the electric field distributions around the free surface and the bubbles were calculated respectively by the Maxwell 2D, and the simulation results were in good agreement with the experimental results.

## Abbreviations

BE: Bubble electrospinning
Co., Ltd.: Limited company
FSE: Free surface electrospinning
MFSE: Modified free surface electrospinning
PE: Polyethylene
PVA: Polyvinyl alcohol
SDBS: Sodium dodecyl benzene sulfonate
SEM: Scanning electron microscopy
wt%: Weight fraction
